# Comparing the effectiveness of continuous subcutaneous insulin infusion with multiple daily insulin injection for patients with type 1 diabetes mellitus evaluated by retrospective continuous glucose monitoring: A real-world data analysis

**DOI:** 10.3389/fpubh.2022.990281

**Published:** 2022-08-25

**Authors:** Guo Keyu, Li Jiaqi, Zhang Liyin, Ye Jianan, Fan Li, Ding Zhiyi, Zhou Qin, Li Xia, Yang Lin, Zhou Zhiguang

**Affiliations:** National Clinical Research Center for Metabolic Diseases, Key Laboratory of Diabetes Immunology, Ministry of Education, and Department of Metabolism and Endocrinology, The Second Xiangya Hospital of Central South University, Changsha, China

**Keywords:** type 1 diabetes mellitus, continuous subcutaneous insulin infusion, multiple daily insulin injection, continuous glucose monitoring, glycemic control

## Abstract

**Objective:**

Regarding the effects and practical application of insulin pumps on patients with type 1 diabetes mellitus (T1DM), the real-world evidence is limited especially concerning the incidence of hypoglycemia. This study aimed to compare the efficacy of continuous subcutaneous insulin infusion (CSII) therapy with multiple daily injection (MDI) therapy on glycemic metrics evaluated by retrospective continuous glucose monitoring (CGM) in Chinese patients with T1DM.

**Methods:**

In total, 362 T1DM Chinese patients from the outpatient department of the Second Xiangya Hospital, Central South University, who underwent intensive insulin therapy and used a retrospective CGM system were included in this retrospective cross-sectional study. Comprehensive analysis of clinical and biological features and retrospective CGM derived-metrics was performed on the 362 enrolled T1DM patients who underwent CSII (*n* = 61) or MDI (*n* = 301) therapy (defined as 4 or more insulin injections per day).

**Results:**

Our findings demonstrated that patients who underwent CSII therapy, compared with those who received MDI therapy, had lower levels of hemoglobin A1c (HbA1c) and fasting blood glucose; moreover, CSII therapy was associated with better glycemic outcomes in terms of increasing time in range (TIR), decreasing time above range (TAR), and achieving CGM-associated targets of TIR ≥70% and TAR <25%. However, patients who underwent CSII therapy did not experience decreasing time below range (TBR), achieving CGM-associated targets of TBR <4%, and reduction of the risk of hypoglycemia as evidenced by comparing TBR and low blood glucose index (LBGI) between the two treatment regimens. The parameters of glycemic variability, such as standard deviation of glucose (SD), mean amplitude glycemic excursion (MAGE), and large amplitude glycemic excursion (LAGE) in T1DM patients who underwent CSII therapy outperformed.

**Conclusion:**

Our results provided further evidence that CSII therapy is safe and effective for management of Chinese T1DM patients, which was confirmed by a lower HbA1c level and better CGM-derived metrics but no demonstration of improvment in the risk of hypoglycemia. To achieve more satisfactory glycemic outcomes through the utilization of CSII therapy for Chinese T1DM patients, a strong physician-patient relationship is essential.

## Introduction

Type 1 diabetes mellitus (T1DM) is a progressive disease as a result of the severe destruction of islet β-cell function, and exogenous insulin is not only essential for more efficiently control of blood glucose levels, but also for T1DM patients to stay in life. (1). In the 100 years since the discovery of insulin, a great progress has been made in our ability to manage T1DM effectively, largely because of the improvements in insulin formulation and delivery (2). Daily multiple insulin injections (MDI) therapy is a well-established intensive therapy, and this basal-bolus therapy is the best therapeutic option for patients with T1DM for a long time, until emergence and development of continuous subcutaneous insulin infusion (CSII) using insulin pumps. An insulin pump mimics the physiological situation by combining a continuous insulin infusion rate to cover the basal insulin requirements with additional bolus deliveries to cover prandial insulin requirements. To date, a number of randomized clinical trials (RCTs) suggested lower levels of hemoglobin A1c (HbA1c) with CSII than with MDI (3). However, whether CSII increase the risk of hypoglycemic events are controversial. With the increasing use of insulin pumps in real-world, the frequency of hypoglycemic events in patients with T1DM has noticeably increased, and the safety of insulin pump therapy has also been concerned (4, 5). Several meta-analyses showing that CSII does not increase the risk of adverse events (maybe with the exception of diabetic ketoacidosis), additionally, and some researches also suggested that a reduction in the incidence of hypoglycemia with pump use compared to MDI (6–10).Although the use of insulin pumps for intensive insulin therapy among patients with T1DM has substantially increased in several developed countries (11), CSII has not been widely used in China (12). A recent multicenter survey of T1DM in Chinese children indicated that only 15.21% of participants received insulin pump therapy. The application of insulin pump therapy varies widely that could be related to the following factors: local economic level, social health insurance, and concerns associating with therapeutic efficacy and hypoglycemic risk in clinical practice.

Regarding the applicability of insulin pump therapy for Chinese patients with T1DM, the real-world evidence is limited, especially concerning hypoglycemia. A recent study examined the effects of CSII therapy on HbA1c level of Chinese patients with T1DM, and found that CSII therapy was associated with a better blood glucose control characterized by a lower HbA1c level. HbA1c level has long been regarded as the gold standard for long-term glycemic control. However, there are several HbA1c-associated limitations, particularly because HbA1c does not always provide an accurate measure of average glucose level and HbA1c does not reflect glycemic excursions (13). Compared with HbA1c, continuous glucose monitoring (CGM) can provide a better and more complete assessment of the glycemic outcomes. In this scenario, HbA1c and CGM-derived metrics are both equally used in T1DM management. CGM has been demonstrated to be clinically valuable, reducing risks of hypoglycemia and hyperglycemia, glycemic variability, and improving patient quality of life for a wide range of patient populations and clinical indications (14–16). Retrospective CGM is a masked device without visual indication of immediate feedback on blood glucose level for patients when they wear CGM, thus, the actual glucose outcomes of interventional measures can be obtained. This feature enables patients less likely to have some unknown behaviors according to the real-time glucose level that may affect the actual glycemic outcome. The present study aimed to compare the efficacy of CSII therapy with MDI therapy on glycemic metrics evaluated by the retrospective CGM in Chinese patients with T1DM.

## Subjects and methods

### Study population

In the present retrospective cross-sectional study, a total of 362 T1DM patients (61 were treated with CSII therapy and 301 with MDI therapy) were admitted to the Second Xiangya Hospital of Central South University (Changsha, Hunan, China) from October 2019 to December 2021 and used a retrospective CGM system (Medtronic plc, Northridge, CA, USA) were enrolled. The study protocol was approved by the Ethics Committees of the Second Xiangya Hospital of Central South University, and it was conducted in accordance with the principles of the Declaration of Helsinki. Written informed consent was obtained from all patients prior to enrollment. The inclusion criteria were as follows: (1) patients who met the 1999 World Health Organization (WHO) diagnostic criteria for diabetes; (2) insulin-dependent diagnosis for T1DM; and (3) patients who were treated with intensive insulin therapy administered by either CSII or MDI, in form of 4 or more insulin injections per day. The exclusion criteria were as follows: (1) utilization of other types of CGM systems during the study period; (2) recently occurrence of complications, including diabetic ketoacidosis, acute infection, chronic infection, surgical complication, trauma, etc.; (3) long-term use of glucocorticoids or immunomodulators; (4) unwilling to wear a CGM device or being allergic to the device; (5) acute and chronic hepatic and renal insufficiency; and (6) presence of autoimmune diseases, such as abnormal thyroid function.

### Data collection

Continuous glucose monitoring was performed using iPro2® as the recorder and an En-lite® glucose sensor (Medtronic plc). The CGM system was placed in the abdominal area and the lateral upper arms according to the manufacturer's instructions. Standard POC capillary blood glucose measurements were carried out by Gold AQ glucometers (Sinocare Co., Ltd., Shanghai, China) for three times/day before breakfast, lunch and dinner to calibrate the CGM system. CGM data were collected from study enrollment until discharge at a week-long study session, and we analyzed average glucose level, estimated HbA1c (eHAb1c) level, glucose variability (calculated as the coefficient of variation, CV; MAGE, mean amplitude glycemic excursion; LAGE, large amplitude glycemic excursion; LBGI, low blood glucose index; HBGI, high blood glucose index), time in range (TIR, 3.9-10.0 mmol/l), time above range (TAR, >10.0 mmol/l), and time below range (TBR, <3.9 mmol/l). The targets were set according to the international consensus guidelines on CGM as follows: TIR ≥ 70%, TAR <25%, TBR <4% and CV <36%, and target HbA1c level of <7.5% was recommended (17).

### Statistical analysis

Normally distributed measurement data were presented as the mean ± standard deviation (SD), and skewed data after normality testing (Shapiro-Wilk test) were expressed as the median and interquartile range (IQR). The independent-sample *t-*test or the Mann–Whitney U test was used to compare differences between groups. Assessment of differences in proportions between two groups was performed by the Chi-square test. A two-tailed *P* < 0.05 was considered statistically significant. SPSS 26.0 software (IBM Corporation, Armonk, NY, USA) was used for statistical analysis.

## Results

### Patients' characteristics

In total, 362 patients with T1DM who were admitted to the outpatient department, Second Xiangya Hospital of Central South University were included in this study, of whom 61 patients received CSII (CSII group) and 301 patients underwent MDI therapy (MDI group). The median duration of time (before enrollment) that the CSII was used by the participants included in the study was 11.5 months. There was a roughly equal proportion of female and male patients (51.9 vs. 48.1%). The mean age was 26 (15, 39) years old. The average duration of T1DM was 2.7 (0.8, 7.0) years. The mean body mass index (BMI) was 20.4 ± 3.2 kg/m^2^. Patients had a mean HbA1c value of 8.0% (7.0%, 9.9%). Patients' demographic and clinical data are presented in Table 1.

**Table 1:** Patients' demographic and clinical characteristics.

	**All patients**	**MDI group**	**CSII group**	***P-*value**
	**(*n =* 362)**	**(*n =* 301)**	**(*n =* 61)**	
Sex (M/F)	188/174	150/151	38/23	0.076
Age (years old)	26 (15,39)	29 (15,42)	25 (15,34)	0.192
Household income^a^ (¥/year)	100,000 (50,000–152,500)	100,000 (50,000–150,000)	100,000 (55,000–190,000)	0.820
Educational status (*n*%)
Less than bachelor's degree	85 (23.5)	73 (24.3)	12 (19.7)	0.072
Bachelor's degree or more	108 (29.8)	82 (27.2)	26 (42.6)	
Unknown	169 (46.7)	146 (48.5)	23 (37.7)	
Diabetes duration (years)	2.8 (0.8,7)	5 (0.8,6.7)	5.9 (1.1,11.1)	0.122
BMI (kg/m^2^)	20.4± 3.2	20.4 ± 3.3	20.1 ± 2.9	0.556
WHR	0.8 (0.8,0.9)	0.8 (0.8,0.9)	0.8 (0.8,0.9)	0.054
Insulin dose (U/kg/day)	0.6 (0.5,0.8)	0.7 (0.5,0.8)	0.6 (0.5,0.7)	0.167
SBP (mmHg)	113 (104,124)	115.4 (104,126)	109.3 (103,116)	0.009
DBP (mmHg)	71± 12	71 ± 12	69 ± 9	0.295
HbA1c (%)	8.0 (7.0,9.9)	8.7 (7.1,10)	8.1 (6.8,8.8)	0.007
HbA1c <7.5% (%)	36.2	32.9	52.5	0.004
FCP (pmol/L)	23.3 (16.5,92.2)	68.3 (16.5,96.8)	43 (16.5,60.8)	0.005
FBG (mmol/L)	8.8 (6.4,11.7)	9.6 (6.7,12.4)	7.5 (4.8,9.4)	<0.001
TC (mmol/L)	4.2 (3.6,4.8)	4.3 (3.6,4.8)	4.4 (3.8,4.8)	0.791
TG (mmol/L)	0.7 (0.6,1.0)	0.9 (0.6,1.1)	1 (0.5,1)	0.637
HDL-c (mmol/L)	1.5 ± 0.4	1.5 ± 0.4	1.5 ± 0.3	0.736
LDL-c (mmol/L)	2.4 (1.9,2.9)	2.5 (1.9,3)	2.5 (2,2.9)	0.546

Data are shown as mean ± SD, median (IQR) and frequency.

BMI, body mass index; WHR, waist-to-hip ratio; MDI, multiple daily insulin injection; CSII, continuous subcutaneous insulin infusion; SBP, systolic blood pressure; DBP, diastolic blood pressure; HbA1c, hemoglobin A1c; FCP, fasting C-peptide; FBG, fasting blood glucose; TC, total cholesterol; TG, triglyceride; HDL-c, high-density lipoprotein cholesterol; LDL-c, low-density lipoprotein cholesterol.

P-values for CSII group vs. MDI group.

a Missing data: 212 (70.4%) in MDI group; 36 (59%) in CSII group.

As shown in Table 1, there was no significant difference in age, gender, duration of disease, and daily insulin dosage between the CSII and MDI groups (All *P* > 0.05). However, fasting C-peptide (FCP) level in the CSII group was significantly lower than that in the MDI group (43.0 vs. 68.3 pmol/L, *P* < 0.001). As for clinical indicators, it was revealed that although the FCP level was lower in the CSII group than that in the MDI group, the HbA1c rate (8.1 vs. 8.7%, *P* = 0.004) and fasting blood glucose (FBG) level (7.5 vs. 9.6 mmol/L, *P* = 0.004) were significantly lower, and the HbA1c rate <7.5% (52.5 vs. 32.9%, *P* <0.001) was significantly higher in the CSII group. Besides, there were no significant difference in BMI, waist-to-hip ratio, and lipid metabolic parameters between the two groups (All *P* > 0.05).

### CGM-derived metrics

**Figure 1:**
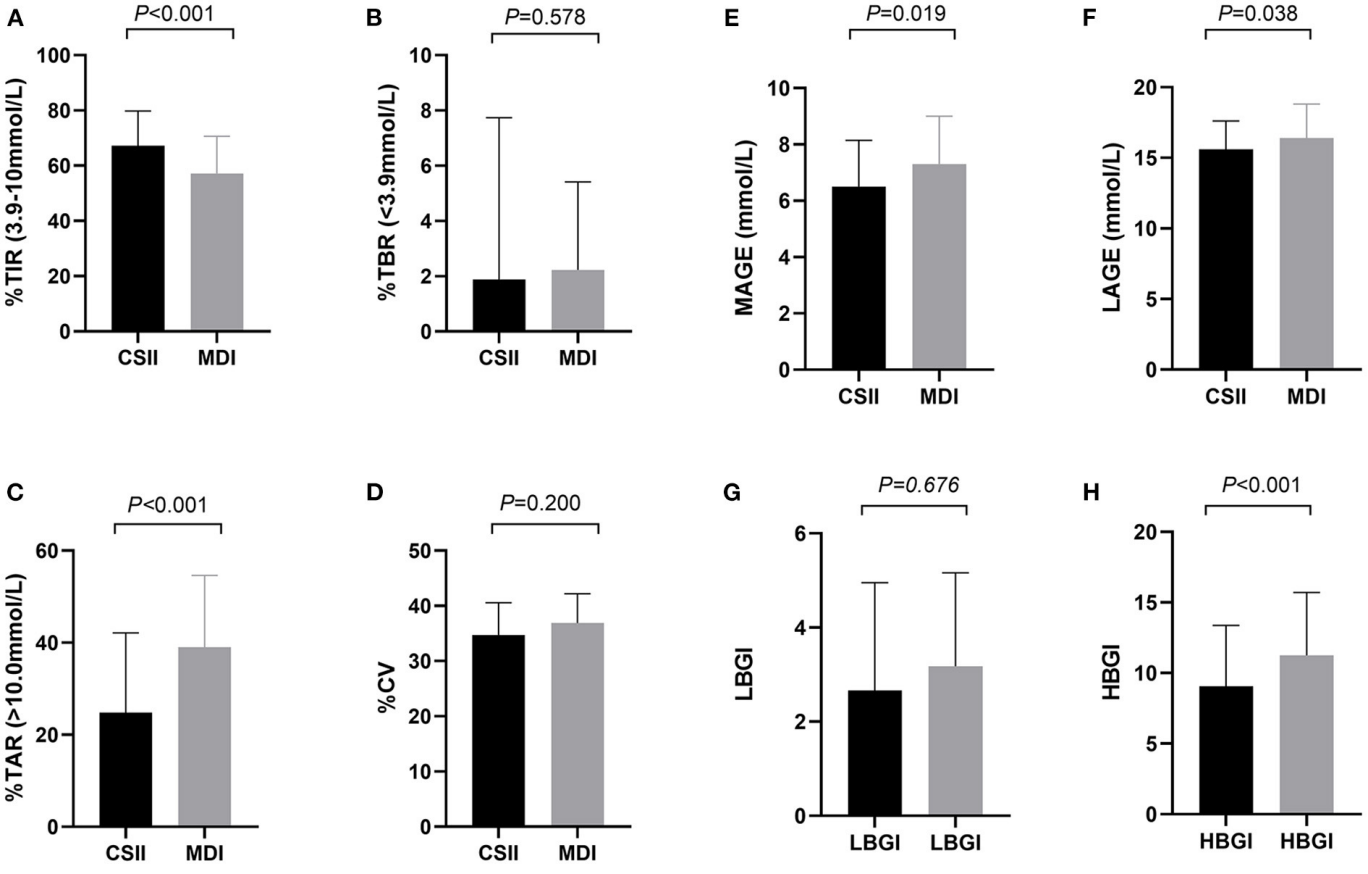
CGM-derived metrics of patients in CSII group and MDI group (*n* = 362). **(A)** TIR, **(B)** TAR, **(C)** TBR, **(D)** CV, **(E)** MAGE, **(F)** LAGE, **(G)** LBGI, **(H)** HBGI. Abbreviations: CGM, continuous glucose monitoring; MDI, multiple daily insulin injection; CSII, continuous subcutaneous insulin infusion; TIR, time in range; TAR, time above range; TBR, time below range; CV, coefficient of variation; MAGE, mean amplitude of glucose excursions; LAGE, mean amplitude of glucose excursions; LBGI, low blood glucose index; HBGI, high blood glucose index. Data are shown as median (IQR). *P* values for CSII group vs. MDI group.

The CGM-derived metrics between the two groups was shown in Table 2. The estimated HbA1c (6.9 vs. 7.5%, *P* < 0.001) reflected the mean blood glucose level (8.3 vs. 9.4 mmol/l, *P* < 0.001), also known as glucose management index, which was significantly lower in the CSII group, and this trend is in line with that of the HbA1c rate. Although no significant difference was found in the CV of glucose between the two groups, the other glycemic variability-related parameters, such as SD (2.9 vs. 3.5 mmol/L, *P* = 0.019), MAGE (6.5 vs. 7.3 mmol/L, *P* = 0.019), and LAGE (15.6 vs. 16.4 mmol/L, *P* = 0.038) of glucose were significantly lower in the CSII group. The TIR was significantly higher in the CSII group than that in the MDI group (44.3 vs. 18.3%, *P* < 0.001). Compared with patients in the MDI group, those patients in the CSII group had significantly lower TAR (24.8 vs. 39.0%, *P* < 0.001) and HBGI (7.5 vs. 11.3, *P* < 0.001). Further investigation indicated that the proportions of time spent within the glucose range of 10.0–13.9 mmol/L (20.0 vs. 24.9%, *P* = 0.019) and >13.9 mmol/L (4.8 vs. 11.1%, *P* < 0.001) were lower in the CSII group. However, no significant difference was found in the TBR and LBGI between the two groups. The results showed that there was no significant difference in the proportions of time spent within the glucose range of 3.0–3.9 mmol/L or < 3.0 mmol/L between the two groups (Figure 1).

**Table 2:** Comparison of CGM-derived metrics between CSII and MDI groups.

	**All patients**	**MDI group**	**CSII group**	***P-*value**
	**(*n =* 362)**	**(*n =* 301)**	**(*n =* 61)**	
eHbA1c (%)	7.4 (6.7, 8.3)	7.5 (6.8, 8.4)	6.9 (6.4, 7.7)	<0.001
%TAR (>10.0mmol/L)	37.6 (21.3, 52.0)	39.0 (22.5, 54.6)	24.8 (17.2, 42.1)	<0.001
TAR <25% (%)	31.8	27.9	50.8	<0.001
%TIR (3.9-10mmol/L)	58.8 (44.9, 73.5)	57.2 (43.2, 70.6)	67.2 (56.4, 79.8)	<0.001
TIR ≥ 70% (%)	22.7	18.3	44.3	<0.001
%TBR (<3.9mmol/L)	2.2 (0.4, 5.6)	2.2 (0.3, 5.4)	1.9 (0.5, 7.7)	0.578
TBR <4% (%)	63.8	63.8	63.9	0.983
Mean glucose (mmol/L)	9.2 (7.9, 10.7)	9.4 (8.0, 11.0)	8.3 (7.4, 9.6)	<0.001
SD (mmol/L)	3.3 (2.7, 4.0)	3.5 (2.8, 4.1)	2.9 (2.4, 3.6)	0.001
MAGE (mmol/L)	6.6 (5.6, 8.9)	7.3 (5.8, 9.0)	6.5 (5.2, 8.2)	0.019
LAGE (mmol/L)	16.4 (13.8,18.6)	16.4 (13.8,18.8)	15.6 (12.4,17.6)	0.038
CV (%)	36.7 (30.1, 42.1)	36.9 (31.0, 42.2)	34.7 (29.7, 40.6)	0.200
CV <36% (%)	46.4	44.2	57.4	0.060
LBGI	3.1 (1.7, 5.1)	3.2 (1.7, 5.1)	2.7 (1.7,5.0)	0.676
HBGI	10.3 (7.0, 15.0)	11.3 (7.4, 15.7)	7.5 (5.2, 12.1)	<0.001

Data are shown as median (IQR) and frequency.

CGM, continuous glucose monitoring; MDI, multiple daily insulin injection; CSII, continuous subcutaneous insulin infusion; eHbA1c, estimated HbA1c; TAR, time above range; TIR, time in range; TBR, time below range; SD, standard deviation of glucose; MAGE, mean amplitude of glucose excursions; LAGE, mean amplitude of glucose excursions; CV, coefficient of variation; LBGI, low blood glucose index; HBGI, high blood glucose index.

P-values for CSII group vs. MDI group.

### CGM-associated target achievement

**Figure 2:**
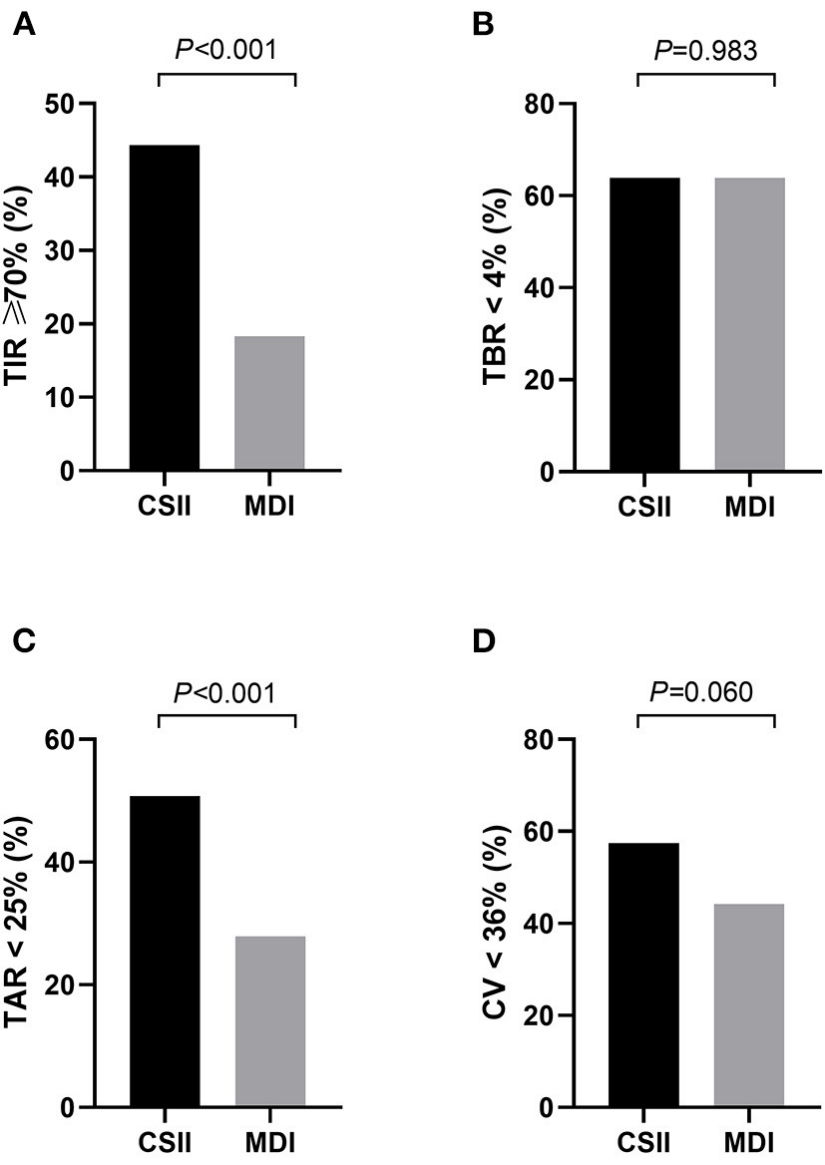
The proportion of patients who achieved the CGM-associated targets in CSII group and MDI group (*n* = 362). **(A)** TIR ≥ 70%, **(B)** TAR < 25%, **(C)** TBR < 4%, **(D)** CV < 36%. MDI, multiple daily insulin injection; CSII, continuous subcutaneous insulin infusion; TIR, time in range; TAR, time above range; TBR, time below range; CV, coefficient of variation. Data are shown as frequency. *P*-values for CSII group vs. MDI group.

The results indicated differences in rates of achieving the CGM targets according to the treatment regimens (Figure 2). Compared with T1DM patients in the MDI group, those patients in the CSII group had a remarkably higher proportion of TAR <25% (50.8 vs. 27.9%, *P* < 0.001), and higher rates of achieving the targets of TIR ≥ 70 (44.3 vs. 18.3%, *P* < 0.001). In contrast, proportion of TBR >4% was comparable between the two groups. The proportion of CV <36% was higher in the CSII group than that in the MDI group, while the difference was not statistically significant (57.4 vs. 44.2 %, *P* = 0.060).

## Discussion

To our knowledge, this is the first study that compared the effectiveness of CSII therapy with that of the MDI therapy on glycemic outcomes evaluated using the retrospective CGM in Chinese patients with T1DM. The main finding of our study is that prescribed CSII therapy was significantly associated with better glycemic outcomes in Chinese patients with T1DM, rather than being correlated with the lower HbA1c, and the better CGM-derived metrics are noteworthy. Among patients with T1DM enrolled in our study, the use of CSII, compared with MDI, resulted in lower levels of HbA1c and FBG, while insulin dose reduction was not significant; moreover, CSII outperformed in increasing TIR, decreasing TAR, and achieving CGM-associated targets of TIR ≥ 70% and TAR < 25%. However, patients who received CSII therapy have not shown decreasing TBR, increasing achieving CGM-associated targets of TBR <4%, and improving the risk of hypoglycemia indicted by comparable LBGI between the two treatment regimens. CGM has traditionally been regarded as the best tool to evaluate short-term glycemic variability in T1DM management, in which the parameters, such as SD, MAGE, and LAGE in patients who received CSII therapy showed better outcomes. Although previous studies have indicated benefits of CSII therapy on glucose variability in patients with T1DM, the real-world evidence is limited and no study evaluated T1DM patients in China using the retrospective CGM to determine glycemic outcomes.

In our study, T1DM patients who received CSII therapy had a significantly better overall glycemic control with the exception of hypoglycemia evaluated by the retrospective CGM. Meta-analyses of the benefits of RCTs demonstrated that CSII therapy compared with MDI therapy mainly exhibited a better HbA1c and a lower risk of severe hypoglycemia (9, 10). In the recent decades, numerous researches have concentrated on the practical application of insulin pumps, and different and even contradictory conclusions in different countries have been reached in a real-world setting (18–21). In the present study, the utilization rate of insulin pumps by T1DM patients was 16.9%, which was relatively consistent with the recently reported findings in China (22, 23). A study that compared CSII and MDI in terms of benefits, safety, and cost-effectiveness showed no significant difference in the level of HbA1c and the incidence of severe hypoglycemia between the two groups, and the cost of CSII treatment was higher (3). The benefits of CSII for long-term glycemic management need to be validated by additional studies in clinical practice. A number of Chinese scholars who concentrated on T1DM patients demonstrated that utilization of insulin pumps is associated with a lower level of HbA1c (12, 24–26). Further cost-effectiveness analysis of CSII therapy vs. MDI therapy also suggested that CSII therapy should be considered as a preferred alternative to MDI therapy for Chinese T1DM patients (12, 26). Our results provided further evidence that CSII therapy is effective for management of Chinese T1DM patients, which was confirmed by a lower HbA1c level and better CGM-derived metrics.

However, there is no evidence indicating the correlation of the utilization of insulin pumps with a lower or a higher hypoglycemia risk based on retrospective CGM-associated metrics in the present study. Correlation of insulin pumps with hypoglycemia has been reported in several RCTs, real-world studies, and meta-analyses, while the results were inconsistent (4, 5, 27–29). Based on the hypoglycemia-related metrics evaluated by the retrospective CGM, the use of insulin pumps is safe as they may not increase or decrease risk of hypoglycemia in our real-world research. Previous real-world studies demonstrated the association of the utilization of insulin pumps with a reduction of adverse hypoglycemic events in patients undergoing CSII therapy, as well as a lower total daily insulin dose compared with patients receiving MDI therapy (11, 30). The daily insulin may have a slightly protective effect on the development of hypoglycemia, which was comparable between the two treatment regimens in our study. Accordingly, it was speculated that daily insulin dose, prandial to total insulin ratio and utilization of rapid-acting insulin analogs can influence hypoglycemia. Therefore, for Chinese T1DM patients, in order to assess the potential role of insulin pumps in reducing the risk of hypoglycemia, the insulin delivery and insulin regimen are equally important.

Chinese T1DM patients undergoing CSII therapy benefited from a better glycemic control compared with those receiving MDI therapy in the present study. In our study, we found an unsatisfactory general rates of target HbA1c level <7.5% and CGM-associated target in Chinese T1DM patients treated with CSII therapy. Therefore, the safety of insulin pumps has been paid intensive attention in China. To achieve desired glycemic targets in T1DM patients undergoing CSII therapy, only dependency on technology is not recommended; the accurate management and utilization of insulin pumps would be the most important steps toward controlling the glycemic outcomes in T1DM patients (31). In addition, an intensive contact between physicians and T1DM patients undergoing CSII therapy is essential to achieve a greater level of knowledge to appropriately carry out insulin dose adjustment for dietary intake, specifically carbohydrate counting, lifestyle factors, and in particular, disease management. An intensive educational program has been developed by our research team *via* a structured T1DM self-management educational system, namely “Type 1 Diabetes Education in Lifestyle and Self Adjustment' (TELSA)”, which could be adapted to medical, social, and cultural practices in China (32). The TELSA program and other intensive educational programs may improve outcomes in Chinese T1DM patients undergoing CSII therapy (33). Moreover, recent studies have demonstrated that intensive insulin therapy of T1DM patients using real-time CGM or flash glucose monitoring is associated with better glycemic outcomes (34–39). In China, the individual version of flash glucose monitoring (Free-Style Libre; Abbott Diabetes Care, Witney, UK) is the most conventional type of CGMs for patients with T1DM caring out of hospitals. A recent research indicated that flash glucose monitoring could be considered as a cost-effective strategy compared with self-monitoring of blood glucose for Chinese T1DM patients receiving insulin therapy (40). Therefore, flash glucose monitoring can be a promising option to improve glycemic control in Chinese T1DM patients undergoing CSII therapy. Further research is required to identify barriers to the effectiveness of insulin pumps for Chinese T1DM patients, in order to provide more targeted measures.

There are some limitations in the present study. The limitations mainly include the lack of the analysis of physical activity, sleep quality, dietary habits, diabetic education, motivation, family support, and mental health factors, influencing the glycemic control of T1DM patients undergoing intensive insulin therapy. Importantly, the observational nature of the study might cause selection bias. Besides, the use of CSII or CGM was highly dependent on patients' and their family member's preferences, economic considerations, or treatment expectancy. In addition, the lack of a long-term study comparing the effects of CSII or MDI therapy on glycemic outcomes in T1DM patients is noteworthy. Therefore, further research should be conducted to eliminate the above-mentioned shortcomings and to confirm our findings.

## Conclusions

In summary, using CSII therapy was significantly associated with better glycemic outcomes compared with MDI therapy in Chinese T1DM patients, which not only would be reflected by the lower HbA1c level, but also by the better outcomes of the most of CGM-derived metrics with comparable hypoglycemia-related parameters. However, the general rates of target HbA1c level <7.5% and CGM-associated target were unsatisfactory in Chinese T1DM patients who underwent both treatment regimens, indicating the necessity of strengthening publicity and educational programs to improve the management of T1DM patients undergoing intensive insulin therapy.

## Data availability statement

The raw data supporting the conclusions of this article will be made available by the authors, without undue reservation.

## Author contributions

YL and ZZ designed the study. GK and LJ conducted the data analysis and drafted the manuscript. GK, ZL, YJ, FL, DZ, ZQ, and LX collected data. All the authors read and approved the submitted version of the manuscript.

## Funding

This study was financially supported by the National Key R&D Program of China (Grant No. 2018YFC2001005).

## Conflict of interest

The authors declare that the research was conducted in the absence of any commercial or financial relationships that could be construed as a potential conflict of interest.

## Publisher's note

All claims expressed in this article are solely those of the authors and do not necessarily represent those of their affiliated organizations, or those of the publisher, the editors and the reviewers. Any product that may be evaluated in this article, or claim that may be made by its manufacturer, is not guaranteed or endorsed by the publisher.
